# Editorial

**DOI:** 10.1093/gbe/evz152

**Published:** 2019-07-19

**Authors:** Laura A Katz, Adam Eyre-Walker

*Genome Biology and Evolution* has turned ten years old. Founded by Bill Martin (University of Duesseldorf, Germany) and Takashi Gojobori (National Institute of Genetics, Japan) as the first open access journal to be owned by an academic society, the Society for Molecular Biology and Evolution, the journal has gone from strength to strength and now receives over 500 manuscripts a year, of which about 60% get published. These vary tremendously in terms of subject matter, organismal focus and methodological approach, but they are bound by a common theme: to understand evolution using genome-scale data.

GBE has published ground-breaking and controversial science over the last ten years. Our most highly cited article is a well-reasoned critique, by Dan Graur (University of Houston, USA) and colleagues, of the ENCODE project’s claim that most of the human genome is functional. Among our other top cited manuscripts are those that focus on transposable elements (i.e. the mobilome), archaeal genomes, plastid origins, non-protein-coding RNAs, *Drosophila* and Lamarckian theory, highlighting the breadth of the journal. However, as with all journals, most papers receive far fewer citations than these, and we take pride in publishing excellent quality manuscripts from emerging fields.

With the combination of decreasing costs of sequencing and increasing availability of open source analytical tools, we expect the field of genome evolution to expand geographically. Currently, a large proportion of the papers we publish come from North America, Western Europe and East Asia, but we are proud of the fact that we published papers from 45 countries in 2018. As part of our strategy to increase submissions from around the world, we are aiming to appoint members of the editorial board from countries that currently have smaller communities of researchers studying genome evolution.

Following the lead set by its parent society SMBE, the journal, under the leadership of Bill Martin, has done much to promote women, and approximately half the editorial board is female. It therefore seems fitting, that after ten years at the helm, Bill Martin has stepped down as Editor-in-Chief and been replaced by not one but two Editors-in-Chief, Laura A. Katz and Adam Eyre-Walker. Laura works on phylogenomics and genome evolution in microbial eukaryotes, and Adam on adaptive evolution and the distribution of fitness effects. We have published a number of articles in GBE and GBE’s sister journal MBE, been on the editorial board of the journals, and we are long-term members of SMBE.

We look forward to continuing to develop the journal, and we are eager to hear suggestions from the community. We welcome analyses of genomic-scale data, theoretical advancements, and method development. We have recently updated the description of article types, and, though we anticipate that most of the manuscripts will be full length articles, there are also opportunities to submit shorter letters, genome reports of data that will be useful to the community, and software/method updates. We are also looking to commission additional reviews and perspectives, the latter allowing you to engage in controversy in our field. We welcome suggestions for debates, which might take the form of two opposing perspectives. In short, manuscripts that help us understand evolution at the genome level are encouraged in a variety of shapes and forms.

GBE is an open access journal, published by Oxford University Press, a not-for-profit publisher. GBE relies on author publication charges to fund its editorial and publishing operations. Any profit that the journal makes is ploughed back into the community through the Society of Molecular Biology and Evolution. However, as a society-owned journal, we believe it is important to publish science irrespective of the ability to pay, and hence we will consider applications for waivers on a case-by-case basis.

Although GBE has had a Twitter account in the past, it has remained underused. However, that is set to change. We have taken on Casey McGrath, who did her undergraduate work with Laura and her PhD with Michael Lynch, as our social media and highlights writer. She has established Facebook and Instagram accounts and will be populating these, along with Tweets, to increase the profile of the papers that GBE publishes. You can follow us @GenomeBiolEvol.

We look forward to receiving your submissions and suggestions as we guide the journal over the coming years.

